# Prognostic Impact of PET/CT-Derived Sarcopenia in Metastatic Breast Cancer Treated with CDK4/6 Inhibitors

**DOI:** 10.3390/jcm15103736

**Published:** 2026-05-13

**Authors:** Selin Cebeci, Zeliha Birsin, Seda Jeral Evinç, Hamza Abbasov, Vali Aliyev, Emir Çerme, Ebru Çiçek, Süheyla Atak, Murat Günaltılı, Murad Guliyev, Nebi Serkan Demirci, Lebriz Uslu Beşli, Özkan Alan

**Affiliations:** 1 Division of Medical Oncology, Department of Internal Medicine, Cerrahpaşa Faculty of Medicine, Istanbul University-Cerrahpaşa, 34320 Istanbul, Türkiye; selin.cebeci@iuc.edu.tr (S.C.);; 2 Department of Nuclear Medicine, Cerrahpaşa Faculty of Medicine, Istanbul University-Cerrahpaşa, 34320 Istanbul, Türkiye; lebriz.uslu@iuc.edu.tr

**Keywords:** metastatic breast cancer, CDK 4/6 inhibitors, sarcopenia, progression-free survival, PET/CT, skeletal muscle index, body composition

## Abstract

**Objective:** This study aimed to evaluate the prognostic significance of positron emission tomography/computed tomography (PET/CT)-derived sarcopenia in patients with hormone receptor-positive, HER2-negative metastatic breast cancer treated with cyclin-dependent kinase 4/6 (CDK4/6) inhibitors. **Methods:** This retrospective single-center study included 77 patients treated between January 2018 and March 2025. Sarcopenia was assessed using skeletal muscle index (SMI) at the L3 level on fluorodeoxyglucose (FDG) PET/CT. Patients were classified as sarcopenic or non-sarcopenic. Clinical, nutritional parameters including body mass index (BMI) and prognostic nutritional index (PNI), and inflammatory parameters including pan-immune inflammation value (PIV) were analyzed. The primary endpoint was progression-free survival (PFS). **Results:** Sarcopenia was present in 35.1% of patients. After a median follow-up of 38 months, sarcopenic patients had significantly shorter PFS compared with non-sarcopenic patients (18 vs. 38 months; HR: 2.37, 95% CI 1.12–4.99, *p* = 0.02, multivariable analysis). In multivariable analysis, sarcopenia, recurrent disease, brain metastasis, and liver metastasis were independent predictors of PFS. No significant association was observed between sarcopenia and overall survival. BMI, PNI, and PIV were not associated with survival outcomes. Toxicity profiles were comparable between groups. **Conclusions:** PET/CT-derived sarcopenia may be a prognostic factor for PFS in patients receiving CDK4/6 inhibitors, whereas conventional nutritional and inflammatory markers are not. These findings support the clinical utility of imaging-based body composition assessment. Prospective studies incorporating functional measures of sarcopenia are warranted.

## 1. Introduction

Breast cancer is the most common malignancy among women, and the most prevalent subtype is hormone receptor (HR)-positive and human epidermal growth factor receptor 2 (HER2)-negative breast cancer. The standard first-line treatment strategy for patients with HR-positive, HER2-negative metastatic breast cancer consists of a cyclin-dependent kinase (CDK) 4/6 inhibitor combined with either an aromatase inhibitor (AI) or fulvestrant. Several factors may influence the response rate to CDK 4/6 inhibitors, including the presence of visceral metastases, endocrine sensitivity, and menopausal status [1].

Changes in body composition, particularly alterations in adiposity distribution and reduced muscle mass, are frequently observed in cancer patients. Sarcopenia is characterized by low muscle strength accompanied by reduced muscle quantity or quality, which can be assessed using imaging techniques such as computed tomography (CT), magnetic resonance imaging (MRI), and dual-energy X-ray absorptiometry (DEXA) [2]. The reported prevalence of sarcopenia among breast cancer patients ranges from 15.9 to 66.9% [3]. According to the World Health Organization (WHO), obesity is defined as a body mass index (BMI) greater than 30 kg/m^2^ [4]. Although widely used in clinical practice, BMI does not distinguish between adipose tissue and lean body mass and may therefore inadequately reflect true body composition in cancer patients. Evidence regarding its prognostic value remains conflicting. In metastatic breast cancer, higher BMI may confer some protection against cachexia and be associated with improved outcomes in certain patients; however, obesity is also linked to chronic inflammation, insulin resistance, altered adipokine profiles, and changes in drug pharmacokinetics that may worsen prognosis [5]. Importantly, BMI does not differentiate between fat and lean mass or between visceral and subcutaneous adiposity [6,7]. Visceral adiposity, in particular, has been associated with poorer outcomes due to its metabolic activity and pro-inflammatory effects [8]. Sarcopenia is highly prevalent among oncology patients and is often exacerbated by cancer-related inflammation and malnutrition, contributing to increased treatment toxicity and poorer survival outcomes [9]. As a result, imaging-based assessment of skeletal muscle mass has gained importance as a more accurate method for evaluating body composition. In this context, sarcopenia, defined by reduced skeletal muscle mass, has emerged as a significant prognostic factor in cancer patients. Given that most oncology patients routinely undergo CT or positron emission tomography/computed tomography (PET/CT) for staging and treatment monitoring, these modalities provide a valuable opportunity for the opportunistic assessment of skeletal muscle mass without additional imaging or radiation exposure. Consequently, cross-sectional imaging not only enables precise body composition analysis but also offers enhanced prognostic insight in clinical practice [6].

Despite the growing recognition of sarcopenia as an important prognostic factor in oncology, data regarding its impact on outcomes associated with CDK4/6 inhibitor therapy remain limited [7,8,9]. While several studies have investigated the prognostic role of sarcopenia in patients with HR-positive/HER2-negative metastatic breast cancer treated with CDK4/6 inhibitors, most have relied on CT-based measurements and have primarily focused on survival outcomes. In addition, limited data are available regarding PET/CT-derived sarcopenia and its integration with systemic inflammatory and nutritional markers. Therefore, further studies are needed to better define the clinical utility of imaging-based body composition assessment in this setting.

In this context, the present study aimed to evaluate the prognostic significance of PET/CT-derived sarcopenia in patients with metastatic HR-positive, HER2-negative breast cancer receiving CDK4/6 inhibitors.

## 2. Materials and Methods

### 2.1. Patients and Data Collection

**Figure 1:**
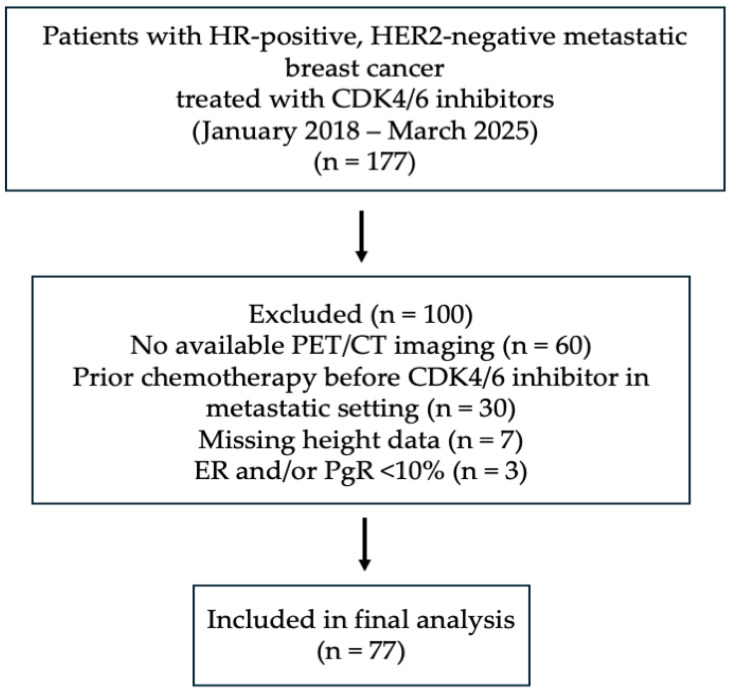
Flow diagram of patient selection.

Medical records of 177 patients treated with CDK4/6 inhibitors (as abemaciclib was not reimbursed in Turkey during the study period, only ribociclib and palbociclib were included) were retrospectively reviewed. This study included female patients (≥18 years) with HR-positive, HER2-negative metastatic breast cancer who received CDK4/6 inhibitor therapy as first- or second-line treatment between January 2018 and March 2025. The majority of patients received CDK4/6 inhibitors in the first-line setting, while a small proportion (*n* = 6) were treated in the second-line setting. HER2 status was defined according to the American Society of Clinical Oncology/College of American Pathologists (ASCO/CAP) guidelines; patients with IHC 0, 1+, or 2+ without gene amplification by in situ hybridization (ISH) were classified as HER2-negative. Estrogen receptor (ER) or progesterone receptor (PgR) positivity was defined as ≥10% by immunohistochemistry. Receptor status was determined based on biopsy results from either the primary tumor or metastatic site, whichever was available. In the metastatic setting, fluorodeoxyglucose (FDG) PET/CT was routinely used for disease evaluation in place of thoracoabdominal CT in most patients in our institution. Sarcopenia was assessed using FDG-PET/CT scans obtained immediately prior to the initiation of CDK4/6 inhibitor therapy. Patients without available PET/CT imaging, those with ER and/or PgR expression <10%, those with missing height data, and those who had received chemotherapy prior to CDK4/6 inhibitor therapy in the metastatic setting were excluded. After applying the criteria, a total of 77 patients were included in the study. A flow diagram illustrating patient selection and exclusion criteria is provided in Figure 1.

### 2.2. Study Variables and Outcome Definitions

Clinical and laboratory data were collected from the institutional electronic medical records system and individual patient files. BMI was available for all included patients. In addition, laboratory parameters reflecting systemic inflammation and potential risk factors for sarcopenia were recorded, including neutrophil, monocyte, and lymphocyte count, serum albumin level, C-reactive protein (CRP), and platelet count.

The pan-immune-inflammation value (PIV) was calculated using the formula: Neutrophil (10^9^/L) × platelet (10^9^/L) × monocyte (10^9^/L)/lymphocyte (10^9^/L) [10]. The prognostic nutritional index (PNI) was calculated using the following formula: 10 × serum albumin (g/dL) + 0.005 × total lymphocyte count (10^9^/L) [11]. The modified Glasgow Prognostic Score (mGPS), based on serum CRP and albumin levels, was also recorded [12]. Data on menopausal status and endocrine sensitivity were also collected and included in the analysis.

The primary endpoint of the study was PFS, and the secondary endpoint was overall survival (OS). For all survival analyses, the initiation of CDK4/6 inhibitor therapy was defined as time zero. For PFS analysis, events were defined as disease progression or death, whichever occurred first. For OS analysis, an event was defined as death from any cause.

Treatment response was evaluated using FDG-PET/CT imaging every three months according to the Positron Emission Tomography Response Criteria in Solid Tumors (PERCIST) [13]. Complete response (CR), partial response (PR), and stable disease (SD) were determined based on the first response assessment, while progression was defined as disease progression occurring at any time during treatment. Treatment-related toxicities were also recorded and graded according to the Common Terminology Criteria for Adverse Events (CTCAE) version 5 [14].

### 2.3. Assessment of Body Composition and Definition of Low Skeletal Muscle Index (SMI)

**Figure 2:**
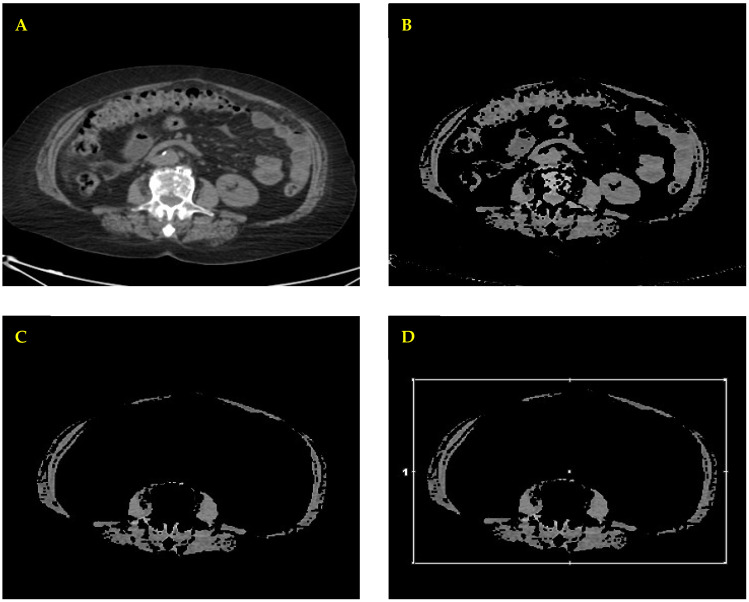
Workflow for skeletal muscle segmentation on axial CT images at the L3 level. Original CT image (**A**), application of Hounsfield unit threshold (−29 to 150) to isolate muscle tissue (**B**), removal of non-muscle structures, including bone marrow, kidneys, vessels, and bowel (**C**), final segmented muscle area used for quantification (**D**).

SMI was assessed using FDG-PET/CT images obtained prior to CDK 4/6 inhibitor therapy, acquired with the same imaging device for all patients to ensure consistency. PET/CT studies were performed using a dedicated PET/CT system (Discovery 710, GE Healthcare, Waukesha, WI, USA). The CT component used for body composition analysis was acquired as a low-dose scan for attenuation correction and anatomical localization. CT acquisition parameters were as follows: tube voltage 120 kV, tube current 200 mAs, and slice thickness 3.75 mm. All images were analyzed using a vendor-specific workstation (AW VolumeShare 7, GE Medical Systems, Buc, France). SMI was calculated at the level of the third lumbar vertebra (L3) by dividing the total skeletal muscle area (cm^2^) by the square of the patient’s height (m^2^) (Figure 2A–D). Manual segmentation at the L3 level was independently carried out by two experienced nuclear medicine specialists blinded to clinical outcomes. All measurements were subsequently reviewed in consensus to minimize interobserver variability.

Receiver operating characteristic (ROC) analyses were conducted to evaluate the discriminative ability of SMI for predicting disease progression. However, SMI did not demonstrate significant discriminative performance for predicting progression (AUC = 0.449, *p* = 0.449), 12-month PFS (AUC = 0.423, *p* = 0.332), or 24-month PFS (AUC = 0.382, *p* = 0.082) (Supplementary Figure S1A). This limited discriminatory performance likely reflects the relatively small sample size and the multifactorial nature of disease progression in metastatic breast cancer. Given the lack of statistically significant ROC-derived thresholds, previously validated SMI cut-off values from the literature (SMI < 41 cm^2^/m^2^) were adopted to define sarcopenia. Although this fixed threshold does not account for sex- or BMI-specific differences, it has been widely used in oncologic studies, allowing for comparability across studies and supporting external validity [15]. In addition, as the study cohort consisted entirely of female patients, sex-specific adjustment was not applicable. Nevertheless, the absence of BMI-specific stratification may have affected the accuracy of sarcopenia classification and should be considered when interpreting the findings.

### 2.4. Ethical Considerations

Access to patient data was restricted to the physicians involved in data analysis and manuscript preparation, in accordance with institutional confidentiality policies. The study was conducted in line with institutional and international ethical standards, including the Declaration of Helsinki. Ethical approval was obtained from the Ethics Committee of Istanbul University-Cerrahpaşa, Cerrahpaşa Faculty of Medicine (approval no: 2026/81, dated 4 March 2026).

### 2.5. Statistical Analysis

Statistical analyses were performed using IBM SPSS Statistics, version 26.0 (IBM Corp., Armonk, NY, USA). Continuous variables were summarized using descriptive statistics and assessed for normality with the Shapiro–Wilk test. Group comparisons were conducted using the Mann–Whitney U or Kruskal–Wallis tests, as appropriate, while categorical variables were compared using chi-square or exact tests. Survival analyses were performed using the Kaplan–Meier method and compared with the log-rank test. Median follow-up time was calculated using the reverse Kaplan–Meier method. Cox proportional hazards regression was used for univariable and multivariable analyses. Given the relatively limited number of events, variable selection for multivariable models was restricted to factors with *p* < 0.200 in univariable analyses and variables considered clinically relevant based on previously published literature to reduce the risk of overfitting. A *p*-value < 0.05 was considered statistically significant. ROC curve analyses were performed to determine the optimal cut-off value of pan-immune inflammation value (PIV) and prognostic nutritional index (PNI). However, the ROC analyses did not demonstrate statistically significant discriminatory ability for each parameter (PNI: AUC = 0.428, *p* = 0.3; PIV: AUC = 0.419, *p* = 0.23) (Supplementary Figure S1B,C). For PNI and PIV, the median value of the study cohort was used as the cut-off point. Missing data were handled using a complete-case analysis approach; therefore, patients with missing key variables were excluded from the relevant analyses.

## 3. Results

### 3.1. Baseline Clinical and Demographic Characteristic

A total of 77 patients were included in the analysis. The median age of the cohort was 58 (range: 34–82) years. The majority of patients had invasive ductal carcinoma (77.9%). The median ER and PgR expression rates were 95% (range: 30–100%) and 60% (range: 0–100%), respectively, and the median Ki-67 index was 21% (range: 3–90%). Patients with de novo metastatic disease accounted for 50.6% of the cohort, while 49.4% had recurrent disease. Most patients were endocrine-sensitive (79.2%). CDK4/6 inhibitors were initiated as first-line treatment in 92.2% of patients, with ribociclib used in 76.6% and palbociclib in 23.4%. The most common metastatic site was bone (70.1%), followed by lung (35.1%) and liver (22.1%). Baseline clinical and demographic characteristics are summarized in Table 1.

Overall, 35.1% of patients were classified as sarcopenic. Patients were stratified into sarcopenic (*n* = 27) and non-sarcopenic (*n* = 50) groups. Significant differences were observed in BMI and the presence of bone metastases (*p* = 0.002 and *p* = 0.008, respectively). Baseline characteristics according to sarcopenia status are presented in Table 2.

### 3.2. Inflammatory and Nutritional Parameters

Systemic inflammatory and nutritional markers, including PNI, PIV, and mGPS were evaluated. In the overall cohort, the median PIV was 259.26 (range 18.34–5890.5). The median PIV was 235.68 (range 38.53–5890.5) in sarcopenic patients and 274.71 (range 18.34–3047.27) in non-sarcopenic patients. When categorized according to the median value (low vs. high), PIV did not differ significantly between the groups (*p* = 0.37).

In the overall cohort, the median PNI was 52.98 (range 31.4–61.85). The median PNI was 54 (31.4–61.85) in sarcopenic patients and 52.5 (38.5–61.8) in non-sarcopenic patients, with no significant difference observed between the groups (*p* = 0.89).

Similarly, no significant differences in mGPS distribution were observed between sarcopenic and non-sarcopenic patients (*p* = 0.49). Inflammatory and nutritional parameters (PNI, PIV, and mGPS) for the overall cohort and according to sarcopenia status are presented in Table 1 and Table 2.

### 3.3. Progression-Free Survival

The median follow-up time was 38 months (95% CI 31–44.9). During the follow-up period, 44 patients experienced disease progression.

**Figure 3:**
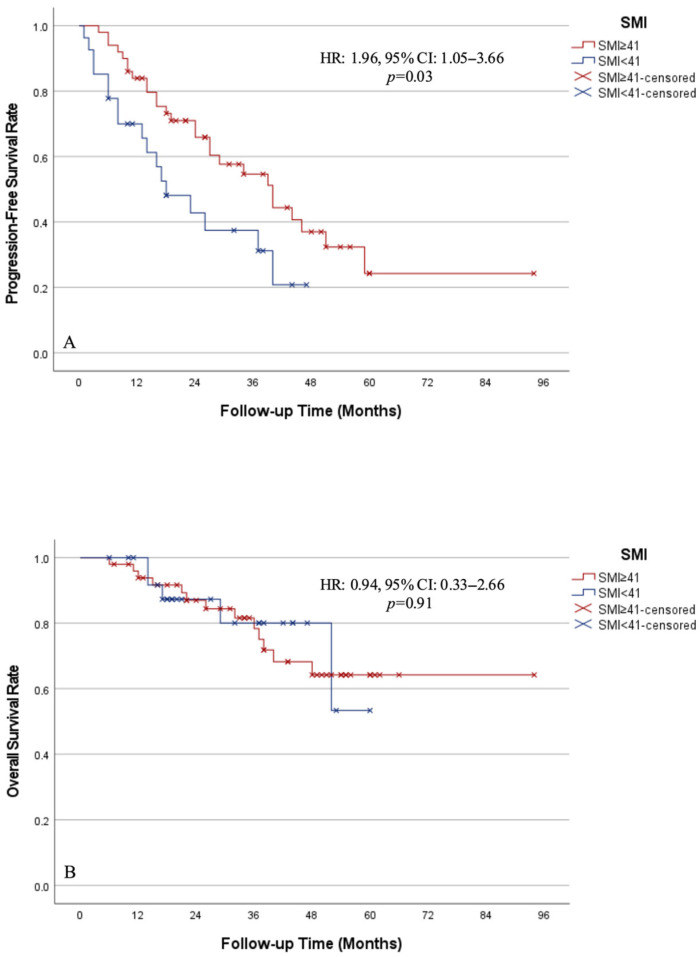
Progression-free (**A**) and overall (**B**) survival according to sarcopenia status.

In the overall cohort, the median PFS was 34 months (95% CI 20.8–47.2) (Supplementary Figure S2A). The median PFS was 18 months (95% CI 8.1–27.9) in sarcopenic patients, and 38 months (95% CI 26.6–53.4) in non-sarcopenic patients. Sarcopenia was associated with a significantly higher risk of disease progression (HR 1.96, 95% CI 1.05–3.66, *p* = 0.03) (Figure 3A).

In the univariable analysis, metastatic presentation (*p* = 0.004), number of metastatic sites (*p* = 0.003), brain metastasis (*p* < 0.001), lung metastasis (*p* = 0.03), and liver metastasis (*p* = 0.006) were significantly associated with PFS, while no other variables showed a statistically significant association. In the multivariable Cox regression analysis, recurrent disease (HR 2.86, 95% CI 1.48–5.52, *p* = 0.002), sarcopenia (HR 2.37, 95% CI 1.12–4.99, *p* = 0.02), brain metastasis (HR 8.77, 95% CI 2.59–29.69, *p* < 0.001), and liver metastasis (HR 2.32, 95% CI 1.09–4.95, *p* = 0.03) were identified as independent predictors of shorter PFS. All results are presented in Table 3.

### 3.4. Overall Survival

During the follow-up period 18 patients died. In the overall cohort, the median OS was not reached. The 24- and 48-month OS rates were 80% and 60%, respectively (Supplementary Figure S2B). Similarly, the median OS was not reached in either the sarcopenic or non-sarcopenic groups (Figure 3B).

In the univariable analysis, the number of metastatic sites (*p* = 0.004), brain metastasis (*p* = 0.004), and liver metastasis (*p* = 0.04) were significantly associated with OS. Age (*p* = 0.06) and bone metastasis (*p* = 0.08) showed borderline associations. No other variables showed a statistically significant association. Sarcopenia was not associated with OS (HR 0.94, 95% CI 0.33–2.66, *p* = 0.91). In the multivariable Cox regression analysis, brain metastasis (HR 9.63, 95% CI 1.85–50.06, *p* = 0.007) and liver metastasis (HR 3.17, 95% CI 1.03–9.75, *p* = 0.04) were identified as independent predictors of shorter OS. All results are presented in Table 4.

### 3.5. Toxicity

Toxicity was observed in 57.1% of the overall cohort. Toxicity rates were similar between sarcopenic and non-sarcopenic patients (57.7% vs. 58.0%, *p* = 0.98). Grade ≥ 3 toxicities were observed in 48.1% of patients, while grade 2 toxicity occurred in 9.1%. Grade 4 toxicity occurred in one patient and was due to neutropenia. No grade 5 toxicity was reported. The most common toxicities were neutropenia, QT prolongation, thrombocytopenia, and anemia. Among grade 3 toxicities, neutropenia was the most frequent (70.5%), followed by thrombocytopenia (9.1%), anemia (9.1%) and QT prolongation (6.8%).

First and second dose reduction and treatment discontinuation rates were similar between groups (*p* = 0.86, *p* = 1, *p* = 0.24, respectively).

Detailed toxicity data are summarized in Supplementary Table S1.

## 4. Discussion

In this retrospective, single-center study, we evaluated 77 patients with metastatic breast cancer treated with CDK4/6 inhibitors in combination with endocrine therapy and investigated the prognostic impact of sarcopenia assessed by PET/CT imaging. Our findings suggest that sarcopenia is an independent predictor of PFS, with sarcopenic patients exhibiting significantly shorter median PFS compared with non-sarcopenic patients. However, these findings should be interpreted with caution, as they may be influenced by potential selection bias, residual confounding, and the relatively small sample size. In contrast, sarcopenia was not associated with OS; however, this finding should also be interpreted cautiously given the limited number of events and the potential for insufficient statistical power. In addition to sarcopenia, recurrent disease, brain metastasis, and liver metastasis were identified as independent predictors of shorter PFS. Notably, conventional nutritional and inflammatory markers, including BMI, PNI, and PIV, were not associated with survival outcomes, highlighting the potential superiority of imaging-based body composition assessment.

These findings are broadly consistent with previous studies reporting an association between sarcopenia and shorter PFS in patients treated with CDK4/6 inhibitors [16,17]. However, our study differs from prior reports in several important aspects. While most previous studies have relied on CT-based measurements, we utilized PET/CT imaging for the assessment of skeletal muscle mass, which may offer a more practical and clinically integrated approach in centers where PET/CT is routinely used. In addition, earlier studies have primarily focused on survival outcomes, whereas our study incorporates a comprehensive evaluation of inflammatory and nutritional markers, including PNI, PIV, and mGPS, enabling a multidimensional assessment of prognostic factors. Furthermore, the predominance of first-line CDK4/6 inhibitor use in our cohort provides a more homogeneous real-world population compared with prior studies that included more heterogeneous treatment settings, which may enhance the clinical interpretability of our findings.

Nutritional status is increasingly recognized as a key determinant of outcomes in oncology; however, conventional measures such as BMI or peripheral blood–based parameters may not adequately reflect body composition. In this context, imaging-based assessment of skeletal muscle mass provides a more precise evaluation. The prognostic relevance of sarcopenia has been well documented across various malignancies. Prado et al. and Shachar et al. demonstrated that, in patients with metastatic breast cancer receiving chemotherapy, sarcopenia was associated with shorter PFS and higher rates of treatment-related toxicity compared with non-sarcopenic patients [18,19]. Similarly, previous studies in metastatic breast cancer, including those evaluating CDK4/6 inhibitors, have generally reported poorer PFS outcomes in sarcopenic patients. Franzoi et al. and Yücel et al. demonstrated markedly reduced PFS in sarcopenic compared with non-sarcopenic patients [20,21]. Consistent with these findings, our study also showed a substantial difference in median PFS (18 vs. 38 months). However, not all studies have demonstrated a significant association, as some reports have shown comparable survival outcomes between groups. In a study by Drittone et al., which included 75 patients treated with CDK4/6 inhibitors, sarcopenic patients had a median PFS of 27 months, compared with 23 months in non-sarcopenic patients and sarcopenia was not found to have a statistically significant impact on PFS or OS [7]. In our study, the median PFS was longer than that reported in previous studies, including the pivotal Paloma and Monaleesa trials [22,23]. Despite a comparable distribution of metastatic sites and a substantial disease burden, as reflected by multiple metastatic sites in many patients, this difference is unlikely to be explained solely by less aggressive disease. Variations between real-world populations and clinical trials, along with the small sample size, may have contributed to these findings. Although SMI did not demonstrate significant discriminatory ability in ROC analysis, it remained significantly associated with PFS in both univariable and multivariable analyses. This apparent discrepancy may reflect methodological differences between ROC and survival analyses. ROC analyses evaluate classification performance at fixed time points, whereas Cox regression models assess longitudinal risk over the entire follow-up period and may therefore better capture the prognostic relevance of variables associated with time-to-event outcomes. Accordingly, the limited discriminatory performance observed in ROC analyses suggests that SMI should not be interpreted as a strong standalone predictive marker. Rather, SMI may represent a prognostic factor that contributes to risk stratification when considered together with established clinical variables. In addition, sarcopenia likely reflects a complex interaction between cancer-related catabolic processes, disease burden, nutritional status, and host-related factors, which may not be fully captured by single-threshold ROC-based classification approaches.

In our study, median OS was not reached in either group. Although preliminary analyses did not demonstrate a statistically significant difference between groups, these results should be interpreted with caution due to the limited number of events and the immaturity of the survival data. In line with these observations, sarcopenia was not significantly associated with OS in regression analysis. Most previous studies have not evaluated OS, likely due to an insufficient number of events. Similarly, Drittone et al. reported comparable OS between sarcopenic and non-sarcopenic patients treated with CDK4/6 inhibitors, and Rier et al. found no association between low muscle mass and OS in patients receiving chemotherapy [7,24]. However, the available evidence remains inconsistent, as some studies have reported significantly shorter OS in sarcopenic patients treated with CDK4/6 inhibitors [16].

Previous randomized trials, including Paloma, Monaleesa, and Monarch have identified endocrine sensitivity, metastatic burden, and visceral disease, particularly liver metastases, as key prognostic factors for PFS [22,23,25]. Consistently, our study found recurrent disease and liver and brain metastases to be independent predictors of progression. Notably, our results further highlight sarcopenia as an additional prognostic factor, with a 2.37-fold increased risk of progression in multivariate analysis (HR: 2.37, 95% CI 1.12–4.99, *p* = 0.02), suggesting that body composition may play a clinically relevant role in treatment outcomes.

In our study, obesity was not associated with PFS or OS. Notably, BMI was significantly higher in the non-sarcopenic group (*p* = 0.002). This finding can be explained by the inherent limitations of BMI, which does not distinguish between fat mass and lean muscle mass. Patients with preserved skeletal muscle mass but increased adiposity may therefore present with a higher BMI without being sarcopenic. This discordance highlights the inadequacy of BMI as a surrogate marker of body composition.

PNI has been associated with survival in breast cancer, reflecting nutritional and immune status [26,27]. However, in our cohort, PNI was not associated with PFS or OS and did not differ between sarcopenic and non-sarcopenic patients. These findings suggest that conventional markers such as BMI and PNI may not adequately capture the prognostic impact of body composition, whereas imaging-based assessment of sarcopenia may be more informative. Similarly, PIV, a marker of systemic inflammation and immune status, was not associated with PFS or OS in our study [28]. Although some reports have linked higher PIV to worse outcomes, others, including a meta-analysis, found no significant association [29]. In addition, PNI and PIV also demonstrated poor discriminatory ability in ROC analysis, further supporting their limited prognostic value in this setting.

In our study, the most common toxicities were neutropenia, QT prolongation, anemia, and thrombocytopenia. Toxicity was observed in 57.1% of the overall cohort, which is slightly lower than the rates reported in pivotal trials [20,21]. Previous studies in the chemotherapy era have shown that sarcopenic patients with metastatic breast cancer experience higher rates of treatment-related toxicity [18,19]. However, more recent studies in the CDK4/6 inhibitor era have reported similar toxicity rates between sarcopenic and non-sarcopenic patients [7,18,21]. Consistent with these findings, our study demonstrated no significant difference in the prevalence of toxicities between sarcopenic and non-sarcopenic patients. Notably, the prevalence of grade 3 toxicities was higher in sarcopenic patients. This finding may be explained by altered pharmacokinetics and reduced physiological reserve in sarcopenic patients. Lower lean body mass may lead to relatively higher effective drug exposure, increasing the risk of hematologic and other toxicities. In addition, sarcopenia is associated with frailty, impaired bone marrow reserve, and systemic inflammation, which may further predispose patients to severe adverse events. In line with these observations, sarcopenia was not significantly associated with CDK4/6 inhibitor type or overall treatment-related toxicity in our cohort, suggesting that its role may be primarily prognostic rather than predictive of treatment tolerance.

This study has several strengths and limitations that should be considered when interpreting the findings. A major strength of our study is the use of PET/CT-based assessment of sarcopenia, which allows for a more precise evaluation of body composition compared with conventional markers such as BMI or laboratory-based indices. To our knowledge, most studies investigating the prognostic impact of sarcopenia in patients receiving CDK4/6 inhibitors have relied on CT-based measurements, while evidence regarding PET/CT-derived sarcopenia in this setting is scarce. In addition, the predominance of first-line CDK4/6 inhibitor use in our cohort and the long median follow-up duration of 38 months enhance the reliability of survival outcomes. Furthermore, the comprehensive evaluation of clinical, inflammatory, and nutritional parameters provides a multidimensional assessment of prognostic factors. However, several limitations must be acknowledged. First, although most patients in our cohort were treated in the first-line setting, a small number of patients (*n* = 6) received CDK4/6 inhibitors in the second-line setting, which may have introduced some degree of heterogeneity. Given the limited number of these patients, subgroup or sensitivity analyses were not performed, as they would not yield statistically robust conclusions. Second, the retrospective and single-center design may introduce selection bias and limit the generalizability of the results. Third, the relatively small sample size may reduce statistical power and contribute to potential overestimation of survival outcomes; moreover, given the limited number of events, the risk of overfitting in multivariable analyses cannot be excluded and should be considered when interpreting the results. Fourth, although PET/CT provides detailed imaging, manual segmentation may be subject to interobserver variability despite consensus review. In addition, interobserver variability was not formally quantified (e.g., by intraclass correlation coefficient), which may affect the reproducibility of the segmentation process. The lack of external validation and the absence of longitudinal assessment of body composition changes over time represent additional limitations, while the use of a fixed SMI cutoff without BMI-specific stratification may have reduced the precision of sarcopenia classification. Moreover, sarcopenia was assessed solely using radiological parameters in our study; however, it is a multidimensional condition that also includes muscle strength and physical performance according to current consensus definitions, which were not evaluated. Additionally, PET/CT-based assessment of sarcopenia is not yet fully validated and requires further standardization.

## 5. Conclusions

In conclusion, our study suggests that sarcopenia may be an independent prognostic factor for progression-free survival in patients with HR-positive, HER2-negative metastatic breast cancer treated with CDK4/6 inhibitors. In contrast, conventional nutritional and inflammatory markers such as BMI, PNI, and PIV were not associated with survival outcomes. These findings indicate that imaging-based assessment of body composition may provide more clinically relevant prognostic information than traditional markers. However, these results should be interpreted with caution given the retrospective design, limited sample size, and potential for residual confounding. Furthermore, sarcopenia is a multidimensional condition, and muscle strength and physical performance are also important components that should be considered in its definition. Larger prospective studies are needed to validate these findings and to further standardize the assessment of sarcopenia in clinical practice.

## Figures and Tables

**Table 1:** Baseline demographic and clinical characteristic of all patients (CDK: Cyclin-dependent kinase, ECOG: Eastern Cooperative Oncology Group, PIV: Pan-immune inflammation value, PNI: Prognostic nutritional index, SMI: Skeletal muscle index) ^a^ Data were missing for one patient; thus, the total evaluable sample was 76 patients, percentages were calculated based on available cases. ^b^ Data were missing for five patients; thus, the total evaluable sample was 72 patients, percentages were calculated based on available cases.

Variables		Results (*n* = 77)
Age	Median	58 (34–82)
	≥65, *n* (%)	24 (31.1)
ECOG, *n* (%)	0	34 (44.2)
	1	39 (50.6)
	2	4 (5.2)
Pathology, *n* (%) ^a^	Invasive ductal	60 (78.9)
	Invasive lobular	3 (3.9)
	Mix	13 (17.1)
Estrogen receptor	Median (range)	95 (30–100)
Progesterone receptor	Median (range)	60 (0–100)
Ki 67 level (%)	Median (range)	21 (3–90)
Metastatic presentation, *n* (%)	De novo	39 (50.6)
	Recurrent	38 (49.4)
Endocrine sensitive, *n* (%)	No	16 (20.8)
	Yes	61 (79.2)
Menopause status, *n* (%)	Pre/perimenopause	22 (28.6)
	Postmenopause	55 (71.4)
Skeletal muscle index, *n* (%)	<41	27 (35.1)
	≥41	50 (64.9)
CDK 4/6 inhibitor, *n* (%)	Ribociclib	59 (76.6)
	Palbociclib	18 (23.4)
Line of CDK 4/6 inhibitor, *n* (%)	First line	71 (92.2)
	Second line	6 (7.8)
Endocrine treatment, *n* (%)	Aromatase inhibitor	61 (79.2)
	Fulvestrant	16 (20.8)
Number of metastatic sites, *n* (%)	1–2	34 (44.2)
	>2	43 (55.8)
Metastatic site, *n* (%)	Cranial	5 (6.5)
	Lung	27 (35.1)
	Liver	17 (22.1)
	Bone	54 (70.1)
Initial treatment response to CDK 4/6 inhibitor, *n* (%)	Complete response	3 (3.9)
	Partial response	55 (71.4)
	Stable disease	12 (15.6)
	Progressive disease	7 (9.1)
PNI	Median (range)	52.98 (31.4–61.85)
PIV	Median (range)	259.26 (18.34–5890.5)
SMI	Median (range)	44.2 (22.6–63.7)
mGPS ^b^	0	47 (65.3)
	1	22 (30.6)
	2	3 (4.2)
Toxicity, *n* (%) ^a^	Absent	32 (42.1)
	Present	44 (57.9)
Progression, *n* (%)	No	33 (42.9)
	Yes	44 (57.1)
Survival, *n* (%)	Alive	59 (76.6)
	Dead	18 (23.4)

**Table 2:** Baseline characteristics by sarcopenia status (AI: Aromatase inhibitor, CDK: Cyclin-dependent kinase, CR: Complete response, ECOG: Eastern Cooperative Oncology Group, IDC: Invasive ductal carcinoma, ILC: Invasive lobular carcinoma, mGPS: Modified Glasgow Prognostic Score, PIV: Pan-immune inflammation value, PNI: Prognostic nutritional index, PD: Progressive disease, PR: Partial response, SD: Stable disease, SMI: Skeletal muscle index). Categorical variables are expressed as number (percentage). Comparisons between groups were performed using the chi-square test or Fisher’s exact test for categorical variables, as appropriate. Bold values represent statistically significant results (*p* < 0.05). ^a^ One non-sarcopenic patient had missing data; percentages were calculated based on available cases (*n* = 49). ^b^ Five non-sarcopenic patients had missing data; percentages were calculated based on available cases (*n* = 45).^c^ One sarcopenic patients had missing data; percentages were calculated based on available cases (*n* = 26).

Variables		Sarcopenic (SMI < 41)	Non-Sarcopenic (SMI ≥ 41)	*p* Value
Age, *n* (%)	<65	22 (81.5)	31 (62)	0.08
	≥65	5 (18.5)	19 (38)	
ECOG, *n* (%)	0	13 (48.2)	21 (42)	0.83
	1	13 (48.2)	26 (52)	
	2	1 (3.7)	3 (6)	
Pathology, *n* (%) ^a^	IDC	21 (77.8)	39 (79.6)	0.97
	ILC	1 (3.7)	2 (4.1)	
	Mix	5 (18.5)	8 (16.3)	
Metastatic presentation, *n* (%)	De novo	14 (51.9)	25 (50)	0.88
	Recurrent	13 (48.1)	25 (50)	
Endocrine sensitive, *n* (%)	No	4 (14.8)	12 (24)	0.34
	Yes	23 (85.2)	38 (76)	
Menopause status, *n* (%)	Pre/peri	8 (29.6)	14 (28)	0.88
	Post	19 (70.4)	36 (72)	
CDK 4/6 inhibitor, *n* (%)	Ribociclib	19 (70.4)	40 (80)	0.34
	Palbociclib	8 (29.6)	10 (20)	
Line of CDK 4/6 inhibitor, *n* (%)	First	26 (96.3)	45 (90)	0.33
	Second	1 (3.7)	5 (10)	
Endocrine treatment, *n* (%)	AI	21 (77.8)	40 (80)	0.82
	Fulvestrant	6 (22.2)	10 (20)	
	>2	14 (51.9)	29 (58)	
	Present	3 (11.1)	2 (4)	
Lung metastasis, *n* (%)	Absent	16 (59.3)	34 (68)	0.44
	Present	11 (40.7)	16 (32)	
Liver metastasis, *n* (%)	Absent	22 (81.5)	38 (76)	0.58
	Present	5 (18.5)	12 (24)	
Bone metastasis, *n* (%)	Absent	3 (11.1)	20 (40)	**0.008**
	Present	24 (88.9)	30 (60)	
Body mass index, *n* (%)	<30	24 (88.9)	27 (54)	**0.002**
	≥30	3 (11.1)	23 (46)	
PIV, *n* (%) ^a^	Low	15 (55.6)	22 (44.9)	0.37
	High	12 (44.4)	27 (55.1)	
PNI, *n* (%) ^b^	<51.7	10 (37)	16 (35.6)	0.89
	≥51.7	17 (63)	29 (64.4)	
mGPS, *n* (%) ^b^	0	18 (66.7)	29 (64.4)	0.49
	1	7 (25.9)	15 (33.3)	
	2	2 (7.4)	1 (2.3)	
Toxicity, *n* (%) ^c^	Absent	11 (42.3)	21 (42)	0.98
	Present	15 (57.7)	29 (58)	
Initial treatment response to CDK 4/6 inhibitor, *n* (%)	CR	0	3 (6)	0.37
	PR	19 (70.4)	36 (72)	
	SD	4 (14.8)	8 (16)	
	PD	4 (14.8)	3 (6)	
Progression, *n* (%)	No	10 (37)	23 (46)	0.45
	Yes	17 (63)	27 (54)	
Survival, *n* (%)	Alive	22 (81.5)	37 (74)	0.46
	Dead	5 (18.5)	13 (26)	

**Table 3:** Univariate and multivariate Cox regression analyses of factors associated with PFS (BMI: Body mass index). Variables found to be significant or close to significance (*p* < 0.200) in univariate analyses and clinically relevant variables were included in multivariate analysis. Bold values represent statistically significant results (*p* < 0.05).

Variables		Univariate (95% CI)	*p* Value	Multivariate (95% CI)	*p* Value
Age	<65	0.82 (0.42–1.59)	0.55		
	≥65				
ECOG	0	1	0.36		
	1	1.24 (0.66–2.32)	0.50		
	2	2.46 (0.71–8.53)	0.2		
CDK 4/6 inhibitor	Ribociclib	1.68 (0.87–3.23)	0.12	1.20 (0.58–2.49)	0.62
	Palbociclib				
Metastatic presentation	De novo	2.52 (1.35–4.72)	**0.004**	2.86 (1.48–5.52)	**0.002**
	Recurrent				
Endocrine sensitive	Yes	1.31 (0.67–2.56)	0.43		
	No				
Menopause status	Pre/peri	0.96 (0.50–1.84)	0.91		
	Post				
BMI	<30	1.10 (0.60–2.04)	0.76		
	≥30				
SMI	≥41	1.96 (1.05–3.66)	**0.03**	2.37 (1.12–4.99)	**0.02**
	<41				
PIV	Low	0.75 (0.41–1.35)	0.33		
	High				
PNI	<51.7	0.77 (0.42–1.40)	0.39		
	≥51.7				
Number of metastatic site	1–2	2.63 (1.38–5.00)	**0.003**	1.91 (0.82–4.47)	0.14
	>2				
Brain metastasis	Absent	7.38 (2.64–20.60)	**<0.001**	8.77 (2.59–29.69)	**<0.001**
	Present				
Lung metastasis	Absent	1.94 (1.06–3.54)	**0.03**	1.52 (0.75–3.06)	0.25
	Present				
Liver metastasis	Absent	2.57 (1.32–4.99)	**0.006**	2.32 (1.09–4.95)	**0.03**
	Present				
Bone metastasis	Absent	1.92 (0.92–3.99)	0.08	1.26 (0.47–3.38)	0.64
	Present				
Toxicity	Absent	0.99 (0.53–1.85)	0.99		
	Present				

**Table 4:** Factors associated with overall survival were identified by univariate and multivariate Cox regression analyses. Variables found to be significant or close to significance (*p* < 0.200) in univariate analyses and clinically relevant variables were included in multivariate analysis. Bold values represent statistically significant results (*p* < 0.05).

Variables		Univariate (95% CI)	*p* Value	Multivariate (95% CI)	*p* Value
Age	<65	2.46 (0.97–6.23)	0.06	2.07 (0.74–5.79)	0.17
	≥65				
ECOG	0	1	0.25		
	1	1.37 (0.50–3.77)	0.55		
	2	3.91 (0.78–19.7)	0.1		
CDK 4/6 inhibitor	Ribociclib	2.05 (0.79–5.29)	0.14	1.87 (0.64–5.46)	0.26
	Palbociclib				
Metastatic presentation	De novo	1.54 (0.60–3.99)	0.37		
	Recurrent				
Endocrine sensitive	Yes	1.54 (0.58–4.11)	0.4		
	No				
Menopause status	Pre	1.39 (0.46–4.23)	0.56		
	Post				
BMI	<30	1.49 (0.59–3.80)	0.40		
	≥30				
SMI	≥41	0.94 (0.33–2.66)	0.91		
	<41				
PIV	Low	0.75 (0.29–1.93)	0.54		
	High				
PNI	<51.7	0.77 (0.30–1.94)	0.57		
	≥51.7				
Number of metastatic site	1–2	8.69 (1.99–37.89)	**0.004**	4.65 (0.84–25.83)	0.08
	>2				
Brain metastasis	Absent	6.98 (1.87–26.01)	**0.004**	9.63 (1.85–50.06)	**0.007**
	Present				
Lung metastasis	Absent	1.61 (0.62–4.19)	0.33		
	Present				
Liver metastasis	Absent	2.83 (1.03–7.75)	**0.04**	3.17 (1.03–9.75)	**0.04**
	Present				
Bone metastasis	Absent	3.72 (0.86–16.19)	0.08	1.59 (0.27–9.53)	0.61
	Present				
Toxicity	Absent	1.12 (0.42–2.99)	0.83		
	Present				

## Data Availability

The original contributions presented in this study are included in the article/Supplementary Material. Further inquiries can be directed to the corresponding author.

## Supplementary Materials

The following supporting information can be downloaded at: https://www.mdpi.com/article/10.3390/jcm15103736/s1, Figure S1: ROC Curve Analysis of SMI (A), PNI (B), and PIV (C) for predicting progression; Figure S2: Progression-free (A) and overall (B) survival in all cohort; Table S1: Treatment-related adverse events Comparisons between groups were performed using the chi-square test or Fisher’s exact test for categorical variables, as appropriate. ^a^ Toxicity grades are presented per patient (maximum grade), whereas individual adverse events are reported per event.

## Abbreviations

AI	Aromatase inhibitor
ASCO/CAP	American Society of Clinical Oncology/College of American Pathologists
BMI	Body mass index
CDK	Cyclin-dependent kinase
CR	Complete response
CRP	C-reactive protein
CT	Computed tomography
CTCAE	Common terminology criteria for adverse events
DEXA	Dual-energy X-ray absorptiometry
ER	Estrogen receptor
HER-2	Human epidermal growth factor receptor-2
HR	Hazard ratio
HR	Hormone receptor
ISH	In situ hybridization
mGPS	Modified Glasgow Prognostic Score
MRI	Magnetic resonance imaging
OS	Overall survival
PD	Progressive disease
PERCIST	Positron emission tomography response criteria in solid tumors
PET/CT	Positron emission tomography/computed tomography
PFS	Progression-free survival
PIV	Pan-immune inflammation value
PNI	Prognostic nutritional index
PR	Partial response
PgR	Progesterone receptor
ROC	Receiver operating characteristic
SD	Stable disease
SMI	Skeletal muscle index
WHO	World Health Organization
