# Supplementary material for: Prognostic Impact of PET/CT-Derived Sarcopenia in Metastatic Breast Cancer Treated with CDK4/6 Inhibitors
Source: J Clin Med. 2026 May 13;15(10):3736. doi: 10.3390/jcm15103736 (PMC13207476; doi:10.3390/jcm15103736)

Supplementary Table 1. Treatment-related adverse events. Comparisons between groups were performed using the chi-square test or Fisher's exact test for categorical variables, as appropriate. [a] Toxicity grades are presented per patient (maximum grade), whereas individual adverse events are reported per event.

**Variables**		**Sarcopenic (SMI<41) n=27 (%)**	**Non-sarcopenic (SMI≥41) n=50 (%)**	***p* value**
Neutropenia		13 (48.2)	25 (50)	0.74
Qt prolongation		1 (3.7)	4 (8)	0.48
Anemia		3 (11.1)	1 (2)	**0.02**
Thrombocytopenia		0	5 (10)	0.09
Nephrotoxicity		1 (3.7)	0	0.16
Diarrhea		0	1 (2)	0.47
Toxicity grade [a]	2	0	7 (14)	0.08
	3	15 (55.6)	21 (42)	
	4	0	1 (2)	
Toxicity grade	<3	0	1 (2)	**0.04**
	≥3	15 (55.6)	22 (44)	
First dose reduction		10 (37)	22 (44)	0.86
Second dose reduction		2 (7.4)	4 (8)	1
Treatment discontinuation		2 (7.4)	1 (2)	0.24

Supplementary Figure 1. ROC Curve Analysis of SMI (A), PNI (B), and PIV (C) for predicting progression

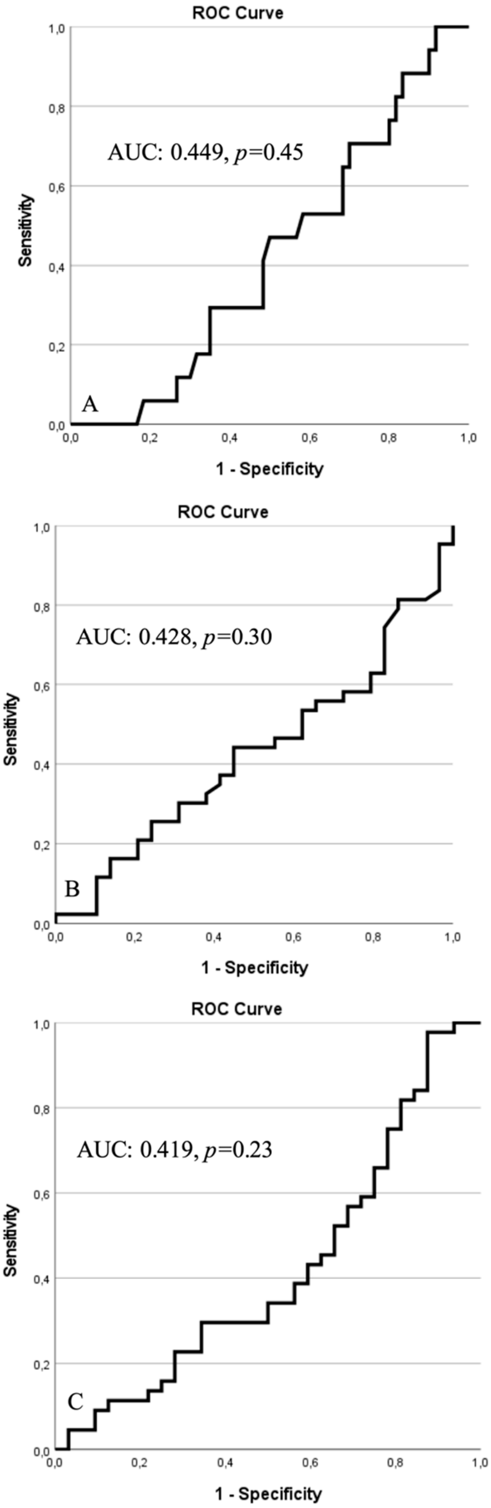

Supplementary Figure 2. Progression-free (A) and overall (B) survivals in all cohort.

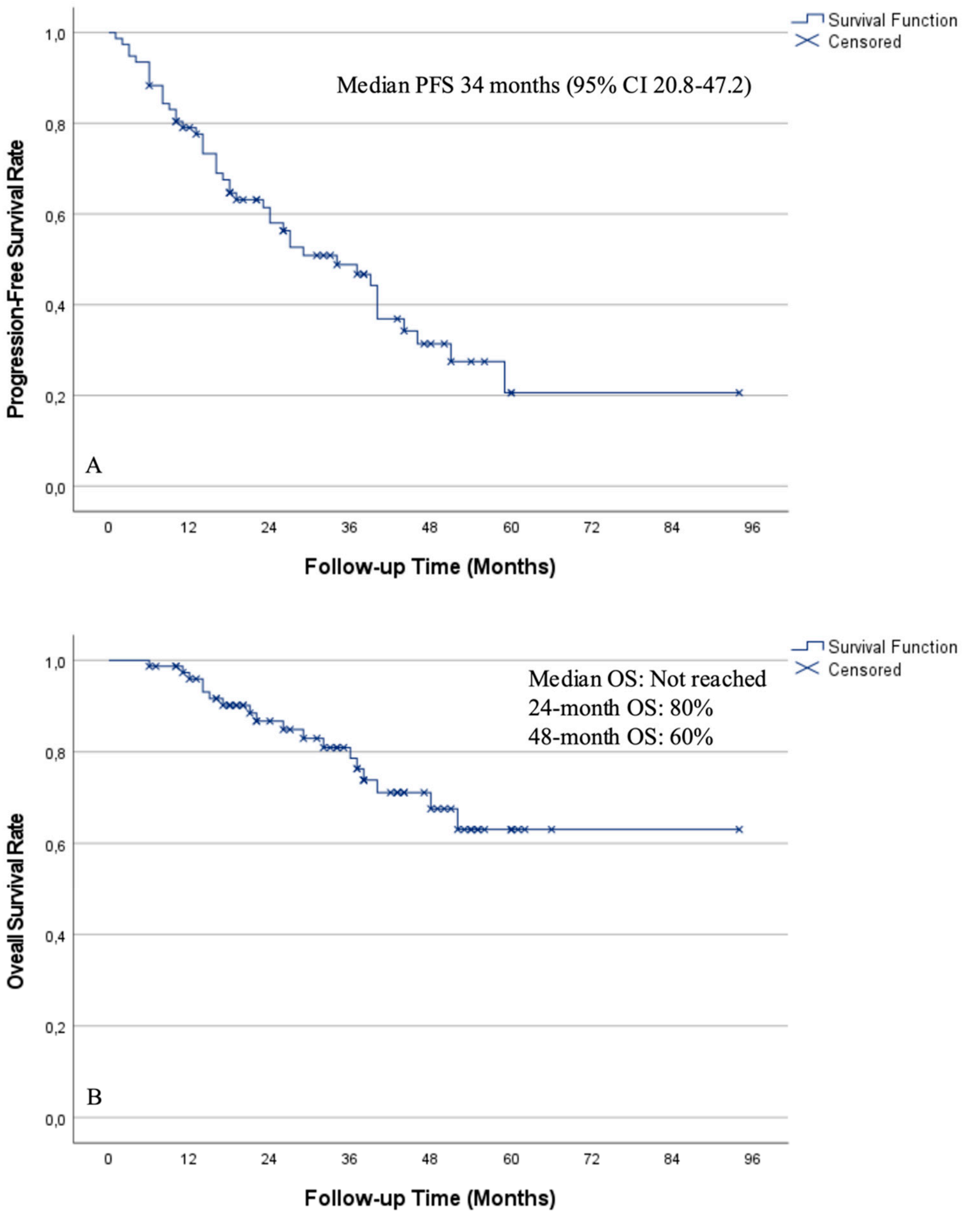

Supplement: Supplementary file 1 [file jcm-15-03736-s001.zip › jcm-4284670-supplementary.pdf]
